# Exploring the Catalytic Mechanism of the RNA Cap Modification by nsp16-nsp10 Complex of SARS-CoV-2 through a QM/MM Approach

**DOI:** 10.3390/ijms23010300

**Published:** 2021-12-28

**Authors:** José Rogério A. Silva, Jaime Urban, Edson Araújo, Jerônimo Lameira, Vicent Moliner, Cláudio Nahum Alves

**Affiliations:** 1 Laboratório de Planejamento e Desenvolvimento de Fármacos, Instituto de Ciências Exatas e Naturais, Universidade Federal do Pará, Belem 66075-110, Brazil; urban@ufpa.br (J.U.); edsonlead@gmail.com (E.A.); lameira@ufpa.br (J.L.); 2 Institute of Biological Sciences, Federal University of Pará, Belem 66075-110, Brazil; 3 BioComp Group, Institute of Advanced Materials (INAM), Universitat Jaume I, 12071 Castellon, Spain

**Keywords:** SARS-CoV-2, nsp16-nsp10, 2′-*O* methylation, catalytic mechanism, QM/MM, TS stabilization, free energy

## Abstract

The inhibition of key enzymes that may contain the viral replication of severe acute respiratory syndrome coronavirus 2 (SARS-CoV-2) have assumed central importance in drug discovery projects. Nonstructural proteins (nsps) are essential for RNA capping and coronavirus replication since it protects the virus from host innate immune restriction. In particular, nonstructural protein 16 (nsp16) in complex with nsp10 is a Cap-0 binding enzyme. The heterodimer formed by nsp16-nsp10 methylates the 5′-end of virally encoded mRNAs to mimic cellular mRNAs and thus it is one of the enzymes that is a potential target for antiviral therapy. In this study, we have evaluated the mechanism of the 2′-*O* methylation of the viral mRNA cap using hybrid quantum mechanics/molecular mechanics (QM/MM) approach. It was found that the calculated free energy barriers obtained at M062X/6-31+G(d,p) is in agreement with experimental observations. Overall, we provide a detailed molecular analysis of the catalytic mechanism involving the 2′-*O* methylation of the viral mRNA cap and, as expected, the results demonstrate that the TS stabilization is critical for the catalysis.

## 1. Introduction

In September 2021, the World Health Organization (WHO) reported over 220 million cases of COVID-19 and over four million fatalities since the beginning of the pandemic [1]. COVID-19 is caused by the severe acute respiratory syndrome coronavirus 2 (SARS-CoV-2), which is an enveloped β-coronavirus with a large, complex positive-sense single-stranded RNA genome [2]. Coronaviruses have one of the largest genomes of all RNA viruses. In particular, the genome of SARS-CoV-2 has ~29,800 bases, which encodes four structural and 16 nonstructural proteins (nsp1-nsp16) that are essential for the lifecycle of this virus [3,4]. Inhibitors of the SARS-CoV-2 virus can be developed for particular targets that play important roles in viral replication. Most eukaryotic cellular and viral mRNAs are modified by the addition of a polyadenine tail at the 3′-terminal and a cap structure at the 5′-end. The RNA cap protects mRNA from degradation by 5′ exoribonucleases, ensures efficient mRNA translation, and prevents recognition of viral RNA via innate immunity mechanisms [5,6,7]. Eukaryotic viruses generally modify the 5′-end of viral RNAs to mimic cellular mRNA structure, which is important for RNA stability, protein translation, and viral immune escape [8]. RNA cap modification contributes to host cell defense as viral RNA lacking 2′-*O* methylation is sensed and inhibited by the interferon-stimulated IFIT-1 protein [9].

**Figure 1:**
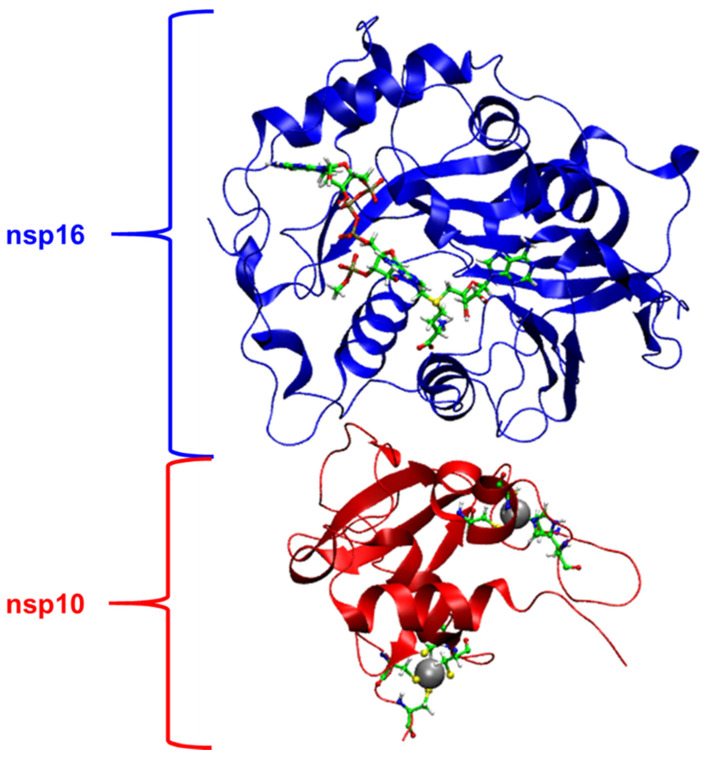
Three-dimensional (3D) structure of nsp16-nsp10 complex (PDB code: 6WKS). On the nsp16, SAM and m7GpppA-RNA are shown as balls and sticks models. On the nsp10, metal centers are highlighted as balls and stick models. The carbon atoms are in green color.

**Figure 2:**
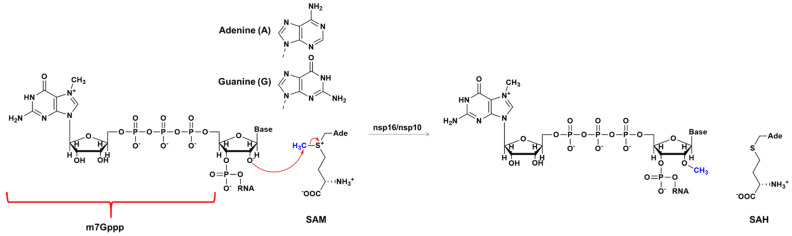
RNA capping mechanisms. The reaction involving m7GpppA(G)-RNA (“p” represents the phosphate group, “m7” is the 7-methylguanosine and “A” (adenine) or “G” (guanine) and SAM.

In this context, nonstructural proteins (nsps) play key roles in RNA capping in coronavirus [10,11,12]. Studies of human and animal coronaviruses have shown that nsp16 is a Cap-0 binding enzyme possessing (nucleoside-2′O)-methyltransferase activity [13]. The activity of nsp16 is enhanced by nsp10, which acts as a cofactor (Figure 1) [14,15]. This nsp16-nsp10 complex methylates the 5′ end of the mRNA, converting the mRNA from an uncapped to the capped form by transferring a methyl group to the first nucleotide on the ribose 2′-*O* moiety of the newly forming mRNA strand. RNA cap modification contributes to host cell defense as viral RNA lacking 2′-*O* methylation is sensed and inhibited by the interferon-stimulated IFIT-1 protein [9]. Recent studies on the nsp16-nps10 protein complex have shown that SARS coronavirus (SARS-CoV) nsp16 methylate RNA cap in the first nucleotide position, usually adenosine [16,17,18]. In the chemical reaction process (Figure 2), the nsp16 transfers a methyl group from S-adenosyl-L-methionine (AdoMet) donor to the unmethylated ribose 2′-*O*, producing RNA-2′-*O*-methylated and S-adenosyl-L-homocysteine (SAH) as a product [8,18]. Previous biochemistry assay has shown that 2′-*O* methyltransferase activity was detected only for m7GpppA-RNA and SAM. On the other hand, m7GpppG-RNA and cap analogs cannot be used as a substrate by 2′-*O*-MTase of SARS-CoV [9]. However, the nsp16 from SARS-CoV-2 has the ability to methylate the GpppA-RNA, GpppG-RNA and GppppA-RNA [19], although, the m7GpppA-RNA is still its best substrate [10,20,21].

The nsp16 is also known as 2′-*O*-RNA methyltransferase (MTase) which is one of the enzymes of the SARS-CoV-2 that is a potential target for antiviral therapy as it is essential for coronavirus replication [8,14]. Many viruses, including all the coronaviruses strains, have virally encoded methyltransferases to disguise and hide viral RNA thus preventing detection by host cell sensing machinery, avoiding recognition and cellular intrinsic defense mechanisms [8]. Nsp16 activity can also help the virus to avoid immune detection and clearance by host immune responses [8]. Experiments in mice with SARS-CoV have shown that the inactivation of this 2′-*O*-MTase activity resulted in a significant reduction of viral replication, reduced weight loss, and limited breathing dysfunction [22]. Understanding the catalytic mechanism of this MTase is important for describing molecular details of SARS-CoV-2 and other RNA virus infections. Recently, Viswanathan and coworkers [18] have demonstrated that nsp16-nsp10 showed remarkable 2′-*O* methyltransferase activity, where the first transcribing nucleotide of the RNA is an adenine, which is always the first nucleotide in the RNA cap synthesis in Coronaviruses. However, it was also observed a remarkable reduction in activity when the first transcribing nucleotide (N1) was changed from the cognate adenine to non-cognate base guanine (the reaction is depicted in Figure 2) [18]. Our previous simulation studies have indicated that the origin of the catalytic effect of methyltransferases is mainly due to electrostatic preorganization [23,24,25,26,27]. The present work evaluates the mechanism of the 2′-*O* methylation of the viral mRNA cap using hybrid quantum mechanics/molecular mechanics (QM/MM) approach [28]. Overall, we aim to understand the determinants of RNA cap modification and describe a general mechanism for 2′-*O* methylation that may be the same for all coronaviruses.

## 2. Results and Discussion

### 2.1. Structural Analysis of MD Simulations

As already stated in the Introduction section, a marked reduction in nsp16 activity was observed when the first transcribing nucleotide (N1) was changed from the cognate adenine to non-cognate base guanine [9,18]. Methyltransferases are a chemically tractable target class for drug discovery [29]. This class of enzymes uses a common bimolecular nucleophilic substitution (S_N_2) methyl transfer mechanism, where S-adenosyl-L-methionine (AdoMet or SAM) usually donates a methyl to nitrogen, oxygen, or carbon atoms [30]. In the present case, nsp16 methylates the 5′ end of the mRNA, converting the mRNA from an uncapped (named as cap-0) to the capped (named as cap-1) form by transferring a methyl group to the first nucleotide, adenosine, on the ribose 2′-*O* position of the newly forming mRNA strand [19]. Interestingly, Benoni et al. [19] showed that nsp16-nsp10 complex can also methyl-ate the first guanosine-5′-triphosphate (GTP) nucleobase of pre-capped mRNA, which is the natural substrate of nsp14. In this study, 100 ns of molecular dynamics (MD) simulations were carried out for nsp16-A and nsp16-G systems to gain insight into the atomistic view of pre-reactive nsp10-nsp16-SAM-m7GpppA-RNA and nsp10-nsp16-SAM-m7GpppG-RNA complexes. The mean distances computed during 100 ns of MD for nsp16-A and nsp16-G are depicted in Supplementary Figures S2 and S3. The mean distances obtained from MD show that Lys46 is positioned to form a hydrogen bond between 2′-OH of the ribose and carboxyl group of Asp130 (see also Supplementary Figure S1 for details). Other important electrostatic interaction occurs between amine group of Lys46 and carboxyl group of Glu203. Particularly, Lys170 is stabilized by Glu203 on the nsp16-A system after 60 ns of MD simulations (Supplementary Figure S2). This interaction is not observed on nsp16-G system (Supplementary Figure S3). In addition, the root-mean-square-deviation (RMSD) was used for measuring the difference between the backbones of nsp16-nsp10 complex were calculated through the simulations with respect to average coordinates structures. On each system, the RMSD plot (Supplementary Figures S2 and S3) displays considerable stability. It should be highlighted that substrates (SAM-m7GpppA(G)-RNA) show high stability during whole 100 ns of MD simulations. Therefore, both nsp16-A and nsp16-G systems exhibit overall conservation of the original conformation, within the exception of the relative position of Lys170 in nsp-16A. Interestingly, during the MD simulation the methyl group of SAM is positioned 3.04 ± 0.15 and 3.08 ± 0.16 Å from the 2′-*O*- of the ribose in nsp16-A and nsp16-G, respectively.

### 2.2. The Methyl Transfer from SAM to Ribose

**Figure 3:**
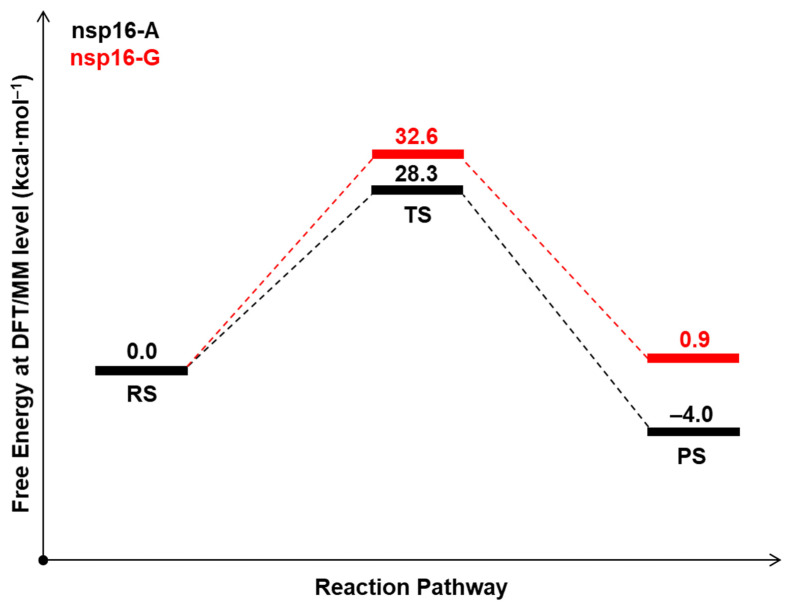
The free energy profile (in kcal·mol^−1^) for the S_N_2 mechanism at M062X-D3/MM level for the nsp16-A (black) and nsp16-G (red) systems.

**Figure 4:**
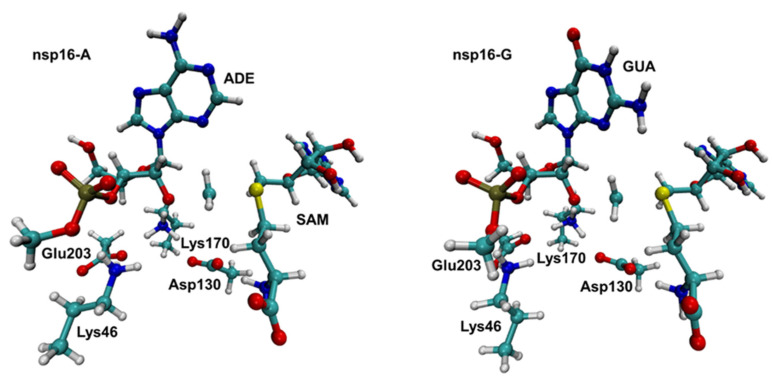
Representative structures of the TS for nsp16-A (**left**) and nsp16-G (**right**) systems. Representative snapshots from QM/MM umbrella sampling molecular dynamics simulations. Note that only atoms in the QM region are shown.

As commented above, experimental data shows that SARS-CoV nsp16-nsp10 complex gave a strong methylation signal on m7GpppA-capped RNA substrate but not on m7GpppG-capped RNA, suggesting that SARS-CoV nsp16-nsp10 functions in a cap sequence-specific manner [18]. Here, we highlight that the crystal structure and biochemical analysis of nsp16 from SARS-CoV-2 has no Mg^2+^ into the high-affinity binding site, which allows a more efficient methyl transfer reaction [31]. Recently, an extensive computational analysis of nsp16-nsp10 complex from SARS-CoV-2 has been performed under same biochemical conditions described here [32]. According to Sk et al., our MD simulations described above shows that both nsp16-A and nsp16-G systems are stable. Then, we used QM/MM and molecular dynamics (MD) simulations to explore the reaction mechanisms involving the methyl transfer from SAM to 2′-OH of ribose of the first transcribing nucleotide of the mRNA cap catalyzed by 2′-*O* methyltransferase (nsp16-A and nsp16-G systems). Computational descriptions of chemical reactions in enzymatic environments are usually based on the selection of distinguished reaction coordinates [33]. As detailed in the Methods section, we have traced the PMFs using the combination of d_1_-d_2_ antisymmetric distance which corresponds to the methyl transfer from SAM to 2-*O*′ of ribose. It is important to mention that a previous study proposed that Lys46 could activate the 2′-OH of the ribose [20]. Then, the 2′-OH of the ribose has to be deprotonated to function as a nucleophile. Figure 3 shows the free energy pathway obtained for the reaction involving the methylation of RNA cap at DFT/MM level, which involves the attack of 2′-*O*- of the ribose on a methyl group bonded to the sulfur atom of the SAM. According to the PMF initially computed at DFTB3/MM level (Supplementary Figure S4), the TS corresponds to a complex (Figure 4), where the SAM and m7GpppA(G)-RNA binds tightly within the active site of the nsp16-nsp10 system. The calculated ΔG^‡^ at DFTB3/MM level are 13.9 and 11.7 kcal·mol^−1^ for the nsp16-A and nsp16-G systems, respectively. At the same QM/MM level, the calculated reaction free energies, ΔG°, are −16.7 and −25.8 kcal·mol^−1^, respectively, showing that both reactions have an exergonic profile. By considering the calculated ΔG^‡^ values at DFTB3/MM, nsp16-A and nsp16-G cannot be distinguished. However, experimental evidence of Viswanathan and coworkers [18] shows that although all components required for methylation are present in both systems, they did not observe methylation of 2′-OH for the nsp16-G system. Then, to improve the description of the QM region in QM/MM simulations, a DFT/MM energy correction was included for describing QM potential (see Methods section). The calculated ΔG^‡^ values using M062X-D3/6-31+G(d,p) for the QM part is 28.3 and 32.6 kcal·mol^−1^ for the nsp16-A and nsp16-G systems, respectively (Table 1). These values are in good agreement with experimental data of other 2′-*O*-methyltransferases [34]. According to Viswanathan and coworkers [18], the positive charge of the sulfur of SAM may repel the purine ring of guanine by a specific interaction with the amino group of guanine. Then, by computing the atomic Mulliken charges (Table 2) on some important atoms of the reaction mechanism, at TS, these values for the S1 atom are 0.03 and 0.07 for the nsp16-A and nsp16-G, respectively. The charge increase of the S_1_ atom occurs due to the effect of the amino group of the purine ring of the guanine, which disfavors the nucleophilic attack from the O_1_ atom of RNA to the C_1_ atom of SAM.

**Figure 5:**
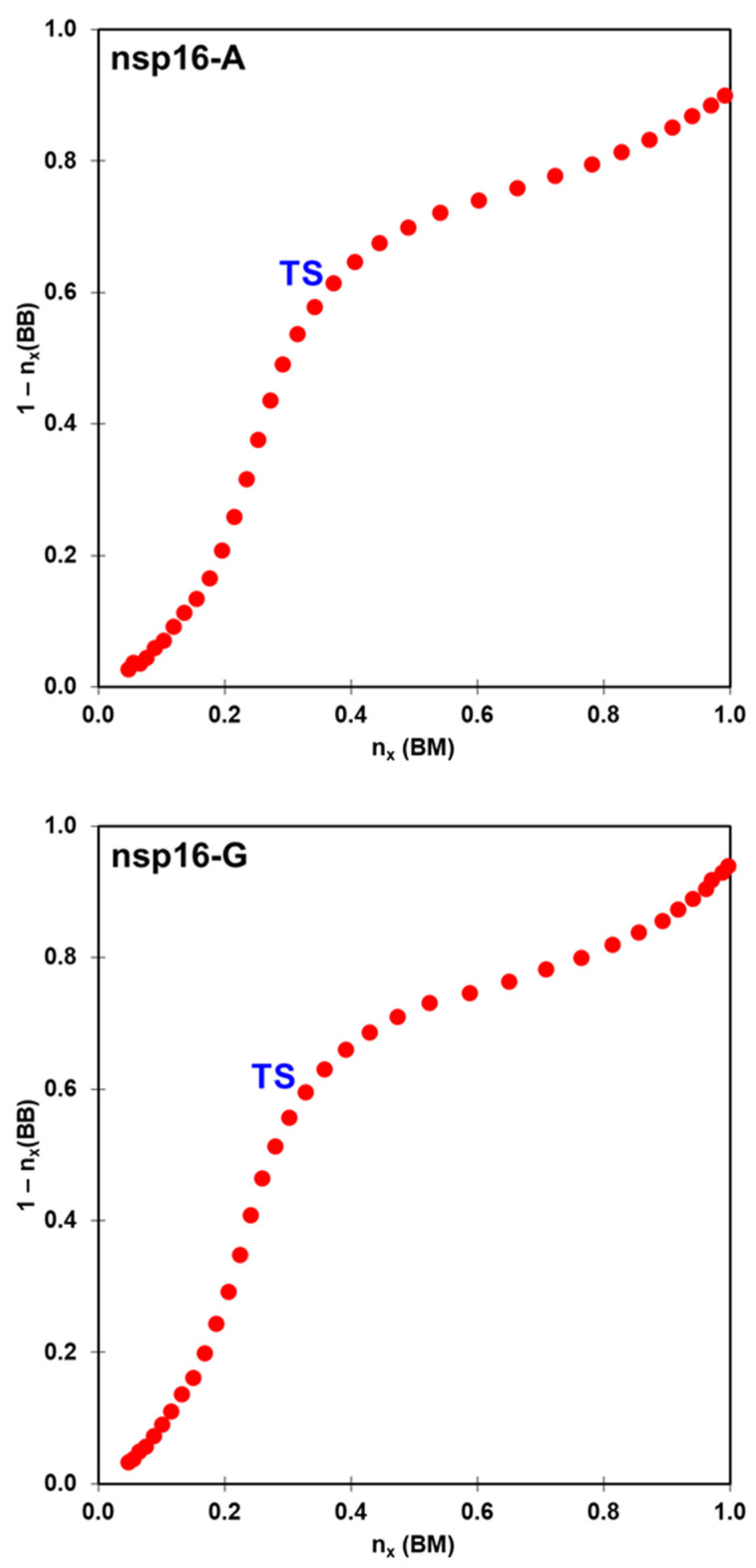
Evolution of O1–C1 bond order, n_x_, for the S_N_2 mechanism of the nsp16-A (**top**) and nsp16-G (**bottom**) systems.

Despite that the energetics obtained at DFTB3/MM level are not in agreement with the experimental observation, the average structures obtained along the reaction path clearly describe a S_N_2 mechanism. In the nps16-A system, at the TS, we obtained d_1_ = 2.05 ± 0.04 Å and d_2_ = 2.36 ± 0.04 Å (Table 2). According to the MOJ plot (Figure 5), this corresponds to bond orders of 0.37 and 0.61 respectively. In the nsp16-G system, at the TS, the d_1_ and d_2_ bonds are equal to 2.10 ± 0.04 Å and 2.31 ± 0.05 Å, respectively. Which correspond to bond orders of 0.33 and 0.60, respectively. For both systems, these orders support the hypothesis that the reaction proceeds via a dissociative S_N_2 mechanism, which means that the leaving group departs well before the TS is reached.

### 2.3. Interaction Energy Decomposition Analysis

**Figure 6:**
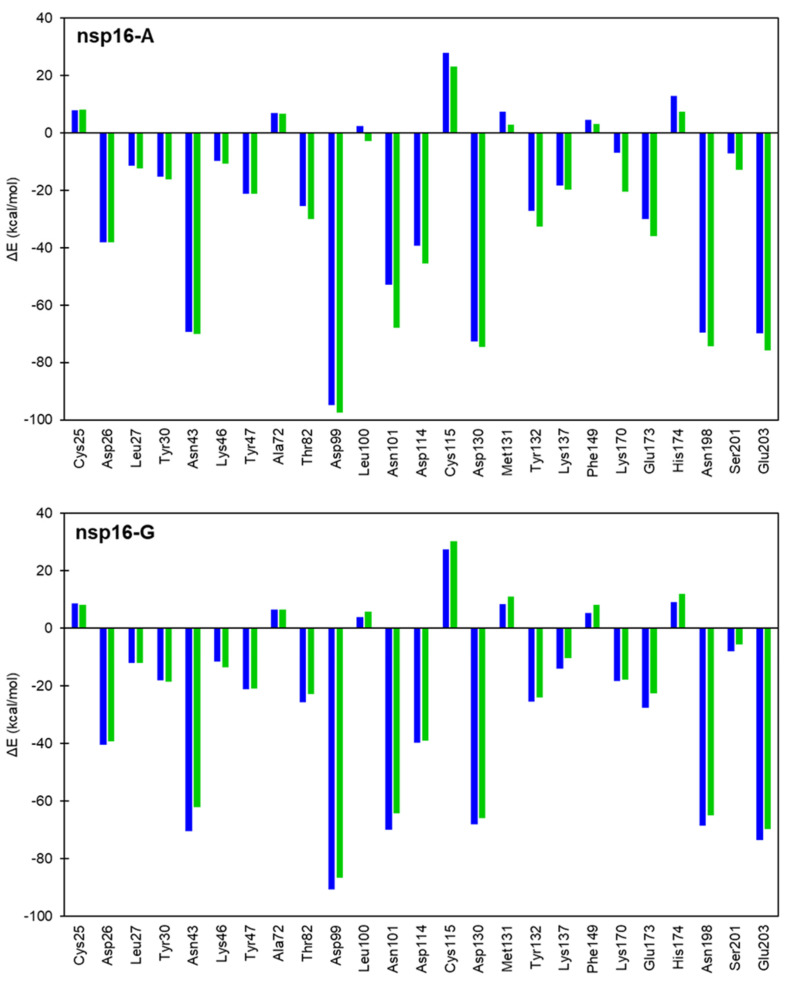
TS (green color) and RS (blue color) stabilization (negative values) or destabilization (positive values) effects of the most relevant active site residues on the nsp16-A and nsp16-G systems at DFTB3/MM level.

The seminal hypothesis of Pauling states that the large rate accelerations for enzymes are due to the high specificity of the protein catalyst for binding the reaction transition state (TS) [35,36]. In principle, the explanation of the catalytic power of enzymes can be achieved by computer-aided methods [37]. This observation allows one to design inhibitors to resemble the structure of a molecular species occurring during the chemical reaction, in particular, transition states. Here, we have explored the total interaction energy on different chemical stages, such as RS and TS, considering energetic changes when a particular amino acid residue is removed from the enzymatic environment, as detailed in the Method section. Here, nsp16 residues that interact with RNA cap and SAM were considered for the interaction energy decomposition analysis. For these calculations, we have defined the QM region as described in the Methods section. The ΔΔE values for each residue reflect the degree of stabilization/destabilization of the RS and TS from the enzymatic environment over the QM part (Figure 6). The results demonstrated how TS is stabilized by amino acid residues around the QM part of nsp16-A (black color) and nsp16-G (red color) systems, where the unfavorable interactions have positive values, while negative values mean favorable interactions (Supplementary Figure S5). It should be highlighted that these values cannot be used to quantitatively compute changes in *k_cat_* produced by Gly-mutation on each residue, since such changes depend on variations in the free energy (ΔG^‡^).

As can be observed in Figure 6, most amino acid residues have strong stabilization effects only on TS of the nsp16-A system. The resultant values computed were −98.7 and 55.5 kcal·mol^−1^, for the nsp16-A and nsp16-G systems, respectively. Particularly, the catalytic mechanism of nsp16 systems involves the tetrad formed by Lys46, Asp130, Lys170, and Glu203 residues [18]. Interestingly, Lys170 and Glu203 have strong beneficial ($\Delta E_{i}^{RS\to TS}$) effects on the nsp16-A system, −13.5 and −6.0 kcal·mol^−1^, respectively. Whereas these residues have deleterious effects on the nsp16-G system, 0.4 and 3.8 kcal·mol^−1^, respectively. Viswanathan and coworkers [18] suggest the influence of Tyr132 for the stabilization of purine base of RNA cap. Our results suggest that it has opposite effects for the TS stabilization. For the nsp16-A system, Tyr132 has a stabilization effect (−5.4 kcal·mol^−1^), while for the nsp16-G system it shows a destabilization effect (1.3 kcal·mol^−1^). Overall, these results are in concordance with the experimental proposal of Viswanathan and coworkers [18], once that mechanism reaction is favorable just for the nsp16-A system.

**Figure 7:**
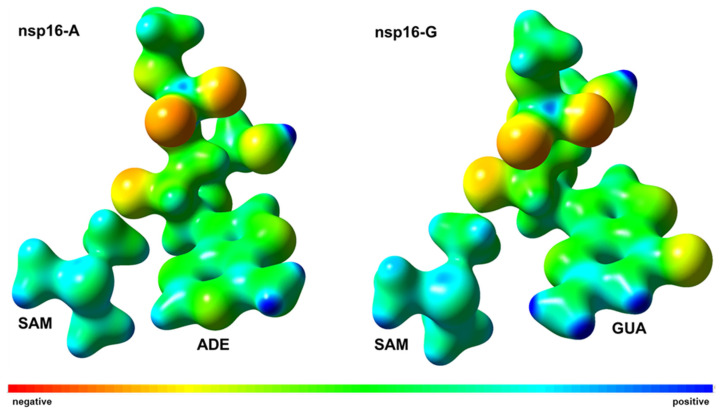
Map of electrostatic potential of the nsp16-A (**left**) and nsp16-G (**right**) systems on the reacting systems at the TS computed at DFT/MM level.

**Figure 8:**
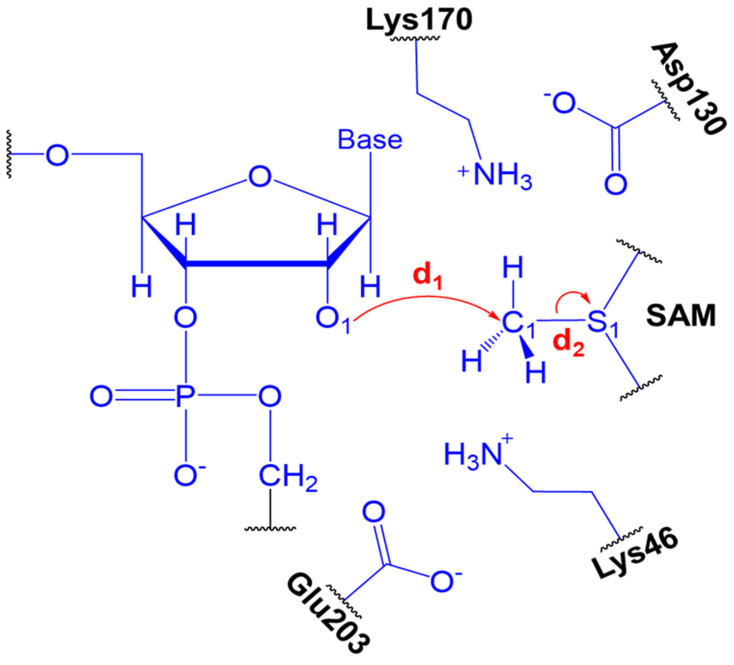
The QM region and reaction coordinate used for QM/MM simulations. This schematic representation involves the ribose of the base, SAM, and the side chain of Glu203, Lys46, Asp130, and Lys170 residues of the active site of the nsp16 protein. The position of the quantum link atoms is indicated as black dots. The atomic coordinates of the QM atoms for TS are included in ESI file. Note that all atoms of SAM were included in QM region during QM/MM simulations.

Viswanathan and coworkers [18] have proposed that a repulsive interaction between sulfur of SAM and the amino group of guanine may repel its purine ring. Then, by computing the map of electrostatic potential (Figure 7) on the reacting systems at the TS under the effect of the protein environment, we suggest that the amino group of guanine can be repelled by the trimethyl sulfonium group of SAM due to positive charge developed around those groups, which is in good concordance to Viswanathan and coworkers [18]. Interestingly, Tyr132 residue has beneficial effects on the nsp16-A system (−5.4 kcal·mol^−1^) whereas it shows deleterious effects on the nsp16-G system (1.3 kcal·mol^−1^). Particularly, π-π stacking interaction can be found between phenyl group of Tyr132 and purine rings of RNA caps. As electronic density is observed on the purine ring of guanine in the nsp16-G system (Figure 8), the effects of π–π stacking interaction are reduced which explains the destabilization effect of Tyr132 in TS of nsp16-G system, as suggested above. Besides, a pyridine base, such as U or C, at N1 position could sterically clash with side chain of Tyr132 and increase the destabilization effects in the TS of nsp16 system.

## 3. Computational Methods

### 3.1. System Setup

To study the methylation reaction involving RNA and MTase we have considered the nsp10-nsp16-SAM complex interacting with RNA, where the crystal structure of the 2-*O*′-Methyltransferase from SARS-CoV-2 (PDB code: 6WKS) [14] was used as the starting point for the computational simulations. Then, the protonation states of the amino acid residues were evaluated by the PROPKA method [38]. The structures of two basis, guanine and adenine, mimicking the RNA and SAM were optimized at HF/6-31G* QM level and the RESP method [39] was used for the partial charges calculations carried out in the Gaussian09 package [40]. The ff14SB [41] and GAFF [42] MM parameters set were used for the protein (nsp10 and nsp16) and ligands (RNA and SAM), respectively. Particularly, as nsp10 is a zinc-binding protein, the MM parameters for the metal centers containing Zn^2+^ ions were obtained from the Zinc AMBER force field (ZAFF) [43]. Then, *tleap* module of the Amber18 package [44] was used to add all missing hydrogen atoms of the protein (nsp10 and nsp16) at pH 7. Each system (nsp10-nsp16-SAM- m7GpppA-RNA and nsp10-nsp16-SAM-m7GpppG-RNA) was immersed in a truncated octahedral cell of TIP3P [45] water molecules, extending 8 Å outside the complex on each side. Here, the nsp10-nsp16-SAM-m7GpppA-RNA (42,483 atoms) and nsp10-nsp16-SAM-m7GpppG-RNA (42490 atoms) systems are named as “nsp16-A” and “nsp16-G”, respectively.

Initially, each solvated complex was energy-minimized by performing a minimization (10,000 steps of each steepest descent and conjugate gradient approach) and then gradually heated up to 300 K over 500 ps of MD simulations while the solute atoms of each system were restrained by applying a harmonic force constant of 100 kcal·(mol·Å^2^)^−1^. Then, the systems were relaxed for 4 ns on seven stages where the restraint constant was gradually decreased until each system be completely released. Finally, 100 ns of MM MD simulations were carried out for each system. In all equilibration and production stages, the particle mesh Ewald (PME) approach computed the long-range Coulomb forces simulations employing a nonbonded cutoff of 10 Å. The hydrogen bonds were restrained by using the SHAKE method [46] during the MD simulations. The MM calculations were performed by the PMEMD module of the Amber18 package [44].

### 3.2. QM/MM Calculations

Despite the development of computer power, as well as parallel computing, it is still extremely challenging to obtain computationally converging sampling of ab initio QM/MM (QM(*ai*)/MM) free energy surfaces in condensed phases (due to a large number of gradient values evaluation) [47]. Herein, the quantum region was described by the 3rd order density-functional tight-binding (DFTB3) semi-empirical method [48] (named as DFTB3/3OB). We choose this semi-empirical potential to compute the activation free energies at a significantly reduced computational cost, allow the application of extensive sampling, and avoid the parameterization required by a more accurate semi-empirical method as Empirical Valence Bond (EVB) [49]. Additionally, the DFTB method has been used to study the catalytic mechanism of methyltransferases [25,50].

As described in Figure 3, the QM part is formed by the side chain of Lys46, Asp130, Lys170, and Glu203, whole SAM, and a piece of RNA containing ribose and adenine (nsp16-A) or guanine (nsp16-G) as purine base. Then, a total of 130 and 131 atoms are included on the QM part of nsp16-A and nsp16-G systems, respectively. The valence of the QM-MM frontier was saturated by using H as link atoms [51]. The total QM charge for each system is equal to “−1”. The rest of the protein and the water molecules were simulated by ff14SB [41] and TIP3P [45] force fields, respectively. The QM/MM calculations were performed by the *sander* module of the Amber18 package [44].

### 3.3. Umbrella Sampling and Free Energy

For each system, equilibrated structures obtained from the 100 ns MD simulations were selected as initial structures for the QM/MM calculations. Potentials of mean force (PMFs) were computed with the umbrella sampling method [52] using the linear combination of inter-atomic distances that corresponds to the methyl transfer from SAM to the negatively charged 2′-OH of ribose of the first transcribing nucleotide of the mRNA cap as the reaction coordinate (RC). As described in Figure 8, the d_1_ describes the nucleophilic attack from the O_1_ atom of the ribose to the C_1_ atom of SAM (bond-forming), while d_2_ describes the bond between C_1_ and S_1_ atoms of SAM (bond-breaking). Each window was computed during 5 ps of equilibration and followed by 50 ps of production with a time step of 1 fs and spaced in steps of 0.10 Å. To restraint the RC during QM/MM umbrella sampling simulations, a harmonic potential with a force constant of 350 kcal·(mol·Å^2^)^−1^ was applied, where the last structure from the previous window was used as the start point for the next umbrella window. Finally, the Weighted Histogram Analysis Method (WHAM) [52] in the WHAM package developed by Grossfield [53] was used to build the probability density and to get the PMFs profiles.

The Pauling bond orders ($n_{x}$) were determined by the bond-making (d_1_) and bond-breaking (d_2_) during all reaction profiles. To understand the real nature of the methyl transfer from SAM to RNA cap SARS-CoV-2 nsp16, a “reaction space” plot based on a More O’Ferrall−Jencks [54] diagram (MOJ) has been plotted to determine the associative or dissociative nature of the S_N_2 reaction. Bond orders were calculated with Equation (1):

(1) $$n_{x}=n_{0}\cdot e^{\left(r_{x}-r_{0}\right)/0.6}$$

In Equation (1), $n_{0}$ means the bond order of the fully formed bond while $r_{0}$ is the equilibrium distance, which was considered equal to 1.45 and 1.77 Å for d_1_ and d_2_ distances, respectively.

### 3.4. High-Level QM/MM Corrections

To improve the DFTB3/MM results, high-level QM corrections were applied at M062X-D3/6-31+G(d,p) level. In this study, the difference in the potential energy barriers obtained from the adiabatic mapping calculations at the DFTB3/MM and M062X-D3/6-31+G(d,p) levels, respectively, were used to include the correction to the 1D-PMF free energy barrier. The optimized coordinates for the QM part along the reaction pathway were then collected and single point energy calculations were carried out in vacuum with *sander* module of Amber18 and Gaussian09 [40] for the DFTB3 and M062X-D3/6-31+G(d,p), respectively. Corrections to the minimum free energy path were applied by subtracting the energy calculated for the QM region using the DFTB3 level and adding the energy from the M062X-D3/6-31+G(d,p) calculations. Similar high-level QM corrections have been applied successfully previously to other enzymatic reactions-catalyzed reactions [55,56]. Besides those approaches can correct the limitations of the lower-level methods, such as energetic calculations of proton affinities. Note that the structures used on the high-level QM corrections were obtained by optimizations of stationary points found on the QM/MM umbrella sampling. Then, PES at the DFTB3 level was obtained on the same umbrella sampling conditions.

For the QM/MM simulations, the effective Hamiltonian is described according to Equation (2):

(2) $${\hat{H}}_{eff}={\hat{H}}_{QM}+{\hat{H}}_{MM}+{\hat{H}}_{QM/MM}$$

where ${\hat{H}}_{QM}$ corresponds to Hamiltonian of the QM region, ${\hat{H}}_{MM}$ is the Hamiltonian of the MM part and ${\hat{H}}_{QM/MM}$ is the hybrid Hamiltonian that includes QM-MM interactions.

From the free energy profile, it should be considered that QM/MM potential can be computed as Equation (3):

(3) $$E_{total}^{protein}=G_{QM}^{gas}+\Delta G_{Xs}+E_{MM}$$

The term $G_{QM}^{gas}$ refers to the gas-phase free energy of the QM part, $\Delta G_{Xs}$ computes the QM/MM interaction free energy and $E_{MM}$ describes the potential of mean force (PMF) for the MM part. It should be considered that term $\Delta G_{Xs}$ has the same value in both high (HL) and low (LL) level of theory, the difference of energy between HL and LL level can be calculated according to Equation (4):

(4) $$E_{total,HL}^{protein}-E_{total,LL}^{protein}=G_{QM,HL}^{gas}-G_{QM,LL}^{gas}$$

In Equation (4), the term $E_{total,HL}^{protein}$ is the corrected free energy, $E_{total,LL}^{protein}$ means the free energy calculated with the umbrella sampling simulations at the DFTB3. The gas-phase energies of the QM part at M062X/6-31+G(d,p) (HL) and DFTB3 (LL) level of theory is represented by $G_{QM,HL}^{gas}$ and $G_{QM,LL}^{gas}$, respectively.

### 3.5. Residual Decomposition Analysis

An energy decomposition method was applied to evaluate how the enzymatic environment stabilizes or destabilizes the transition state (TS) in the catalyzed reaction of SARS-CoV-2 nsp16-nsp10. As this approach has been described in detail elsewhere [57,58,59,60] here only the most relevant equations are presented.

The energetic contribution of an individual residue on the total energy of a particular structure is obtained by the difference of energies when this particular residue is present (*i* state) and when it is mutated to Gly residue (*i* − 1 state) [57] according to Equation (5):

(5) $$\Delta E_{i}=\left[E_{i}^{QM}+E_{i}^{QM/MM}\right]-\left[E_{i-1}^{QM}+E_{i-1}^{QM/MM}\right]$$

In this equation, each term in brackets means the energy changes of the subsystem treated by the QM level in the presence of the MM environment and the interaction energy between the QM and MM part. Next, the deleterious/beneficial effects in going from RS to TS, for each residue, were calculated by Equation (6):

(6) $$\Delta E_{i}^{RS\to TS}=\Delta E_{i}^{TS}-\Delta E_{i}^{RS}$$

Here, the average values of $\Delta E_{i}^{RS\to TS}$ were computed employing 400 snapshots from QM/MM umbrella sampling with reaction coordinates corresponding to the RS and TS. It should be observed that the partition between QM and MM subsystems applied in these computations was different than that used to run the QM/MM umbrella sampling calculations. In particular, the sidechain of residues Lys46, Asp130, Lys170, and Glu203 are not included in the QM subsystem.

## 4. Conclusions

In this study, we have used QM/MM approach to evaluate the reaction mechanisms of the mRNA cap catalyzed by 2′-*O* methyltransferase. Our results suggest that the reaction proceeds via a dissociative S_N_2 mechanism. The calculated ΔG^‡^ correspond to 28.3 and 32.6 kcal·mol^−1^ for the nsp16-A and nsp16-G systems, respectively. These values agree with experimental data for other 2′-*O*-methyltransferases and may explain the remarkable reduction in activity when the nucleotide was changed from adenine to guanine in nsp16 systems. It is important to point out that the enzyme uses key active site interactions that optimally stabilize transition state during the reaction, which explain the reduction in the activation free energy for nsp16-A in comparison with nsp16-G. In particular, the catalytic mechanism of nsp16 systems involves the tetrad formed by Lys46, Asp130, Lys170, and Glu203 residues, where it was observed strong beneficial effects on the nsp16-A system. Additionally, Lys170 and Glu203 have strong beneficial effects on the nsp16-A system, −13.5 and −6.0 kcal·mol^−1^, respectively. Whereas these residues have deleterious effects on the nsp16-G system, 0.4 and 3.8 kcal·mol^−1^. These values demonstrate that substrate makes key intermolecular interaction with residues in active site of enzyme and such interactions stabilize the TS more in nsp16-A than in nsp16-G system. Finally, our QM/MM study complement the structural investigation reported by Viswanathan and coworkers [18].

## Figures and Tables

**Table 1:** Activation-free energy (∆G^‡^) and reaction-free energy (∆G°) for nsp16-A and nsp16-G. DFTB3/MM and its corrected value at M062X-D3/6-31+G(d,p) level are presented. All values in kcal·mol^−1^.

System	ΔG^‡^ (DFTB3/MM)	ΔG° (DFTB3/MM)	ΔG^‡^ (M062X/MM)	ΔG° (M062X/MM)
nsp16-A	13.9	−16.7	28.3	−4.7
nsp16-G	11.7	−25.8	32.6	0.9

**Table 2:** Average key distances (Å) and partial charges (in atomic units) of key atoms involved during the S_N_2 mechanism of the nsp16-A and nsp16-G systems obtained from DFTB3/MM umbrella sampling calculations. Standard deviations are shown in parenthesis.

	nsp16-A			nsp16-G		
	RS	TS	PS	RS	TS	PS
Distances (Å)						
d(O_1_–C_1_)	2.76 (0.04)	2.05 (0.04)	1.47 (0.03)	2.84 (0.05)	2.10 (0.04)	1.46 (0.03)
d(C_1_–S_1_)	1.85 (0.04)	2.36 (0.04)	3.18 (0.04)	1.84 (0.04)	2.31 (0.04)	3.27 (0.04)
Charges (a.u.)						
O_1_	−1.08 (0.03)	−0.80 (0.03)	−0.42 (0.02)	−1.10 (0.02)	−0.83 (0.03)	−0.42 (0.02)
C_1_	−0.26 (0.04)	−0.11 (0.02)	0.00 (0.02)	−0.25 (0.04)	−0.12 (0.02)	0.02 (0.02)
S_1_	0.35 (0.03)	0.03 (0.03)	−0.20 (0.03)	0.36 (0.03)	0.07 (0.03)	−0.18 (0.02)

## Data Availability

Not applicable.

## Supplementary Materials

The following supporting information can be downloaded at: https://www.mdpi.com/article/10.3390/ijms23010300/s1.
