# Supplementary material for: Exploring the Catalytic Mechanism of the RNA Cap Modification by nsp16-nsp10 Complex of SARS-CoV-2 through a QM/MM Approach
Source: Int J Mol Sci. 2021 Dec 28;23(1):300. doi: 10.3390/ijms23010300 (PMC8745711; doi:10.3390/ijms23010300)
Supplement: Supplementary file 1 [file ijms-23-00300-s001.zip › ijms-1467175-supplementary.pdf]

Exploring the Catalytic Mechanism of the RNA cap modification by nsp16-nsp10 complex of SARS-CoV-2 through QM/MM approach

Supporting Information

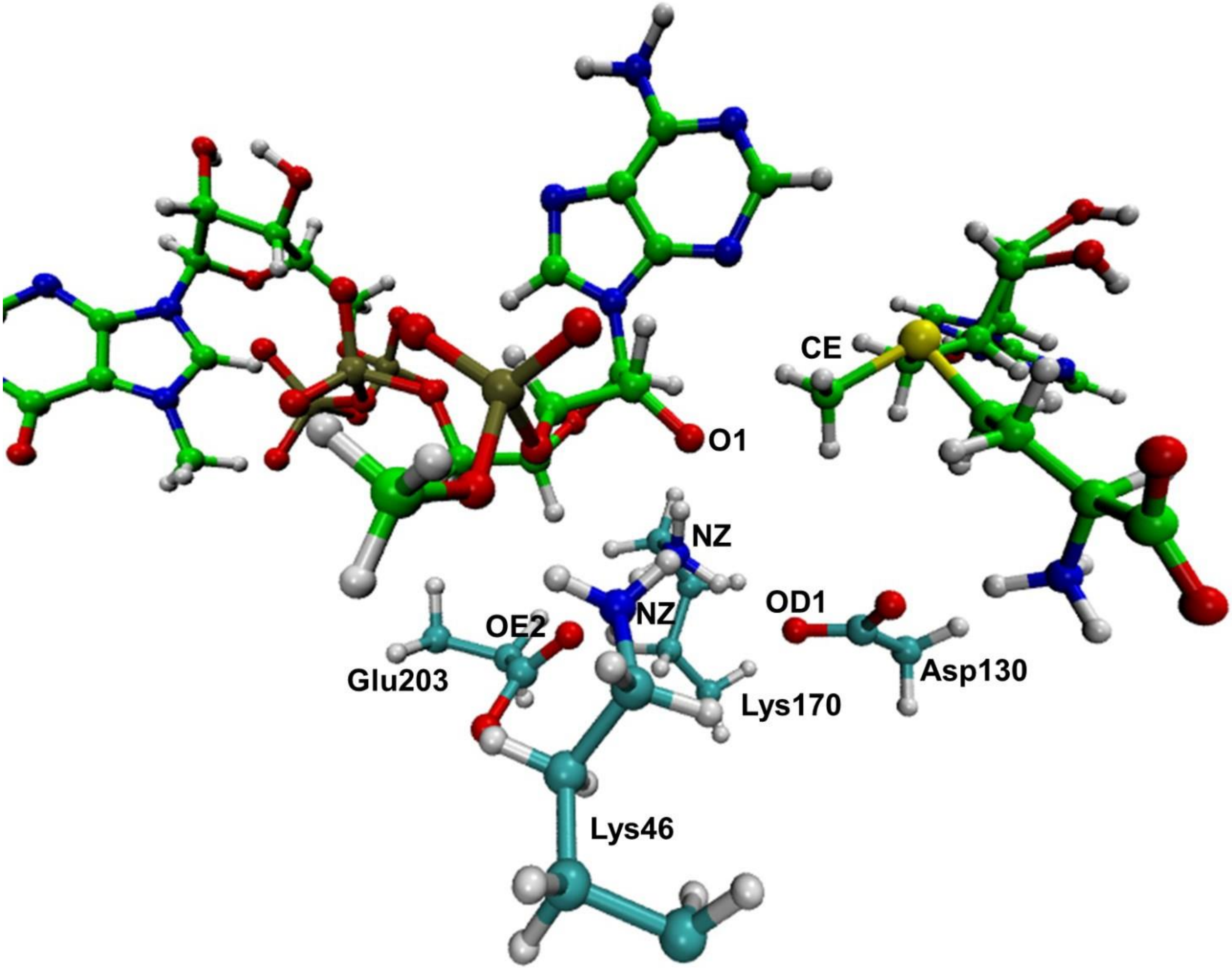

Supplementary Figure S1. A representative snapshot of 100 ns MD simulation for the active site of nsp16-A system. The same atoms label is used for the nsp16-G system.

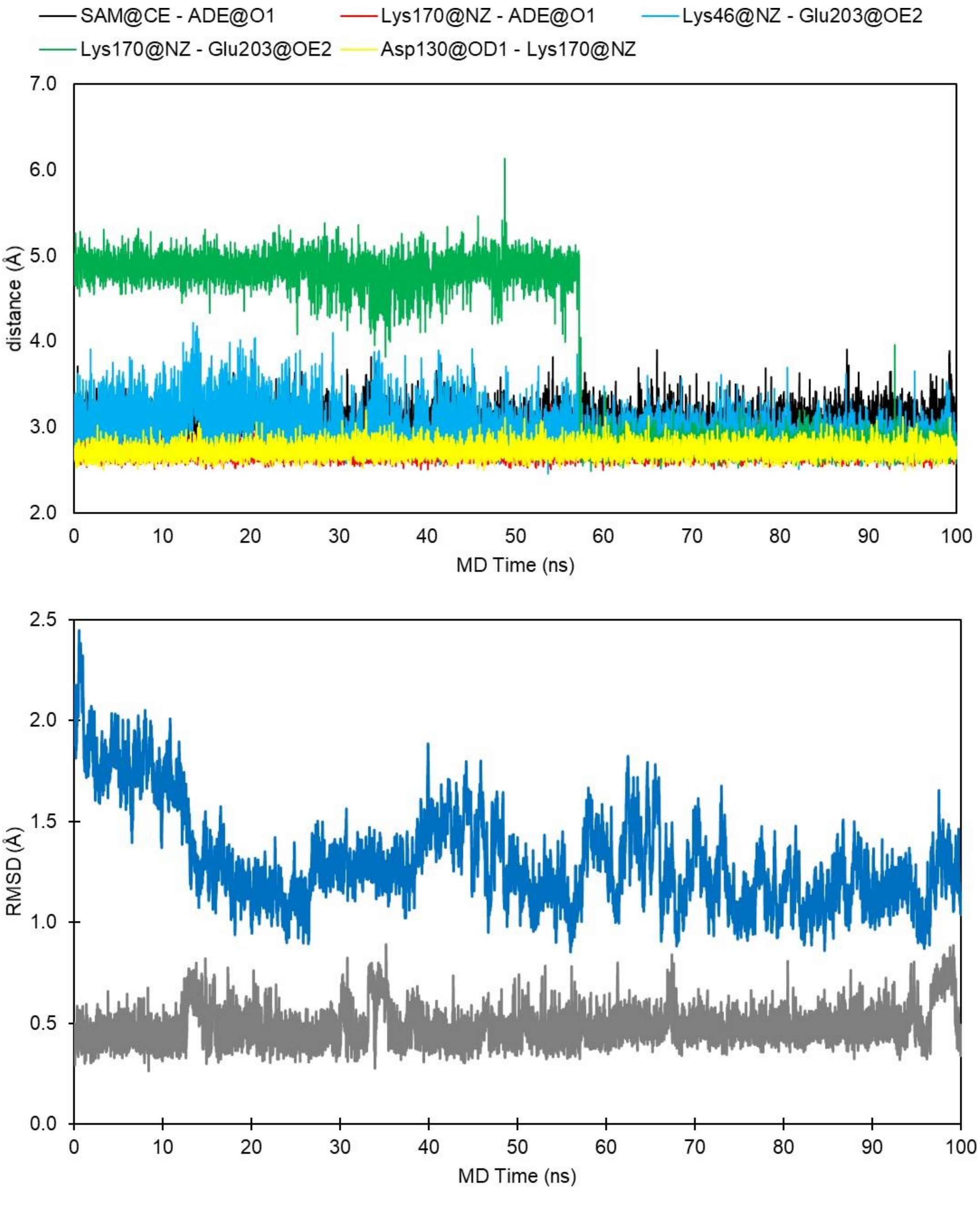

Supplementary Figure S2. On top, plot of main interactions observed between nsp16-A and SAM- m7GpppA-RNA. On bottom, RMSD values for backbone atoms of nsp10-nsp16 system (blue) and SAM-m7GpppA-RNA (gray). A total of 100 ns of MD simulations were considered.

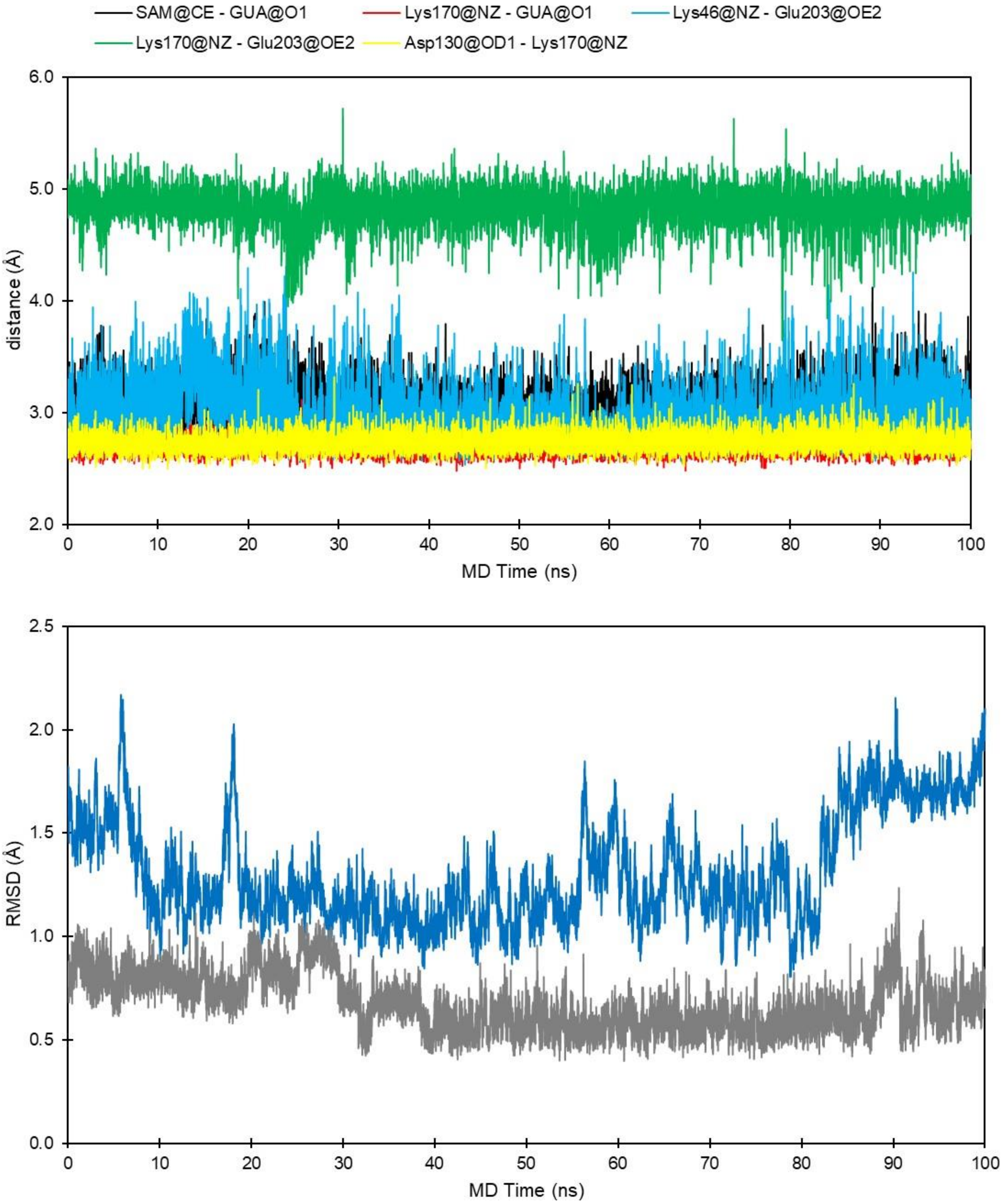

Supplementary Figure S3. On top, plot of main interactions observed between nsp16-G and SAM- m7GpppG-RNA. On bottom, RMSD values for backbone atoms of nsp10-nsp16 system (blue) and SAM-m7GpppA-RNA (gray). A total of 100 ns of MD simulations were considered.

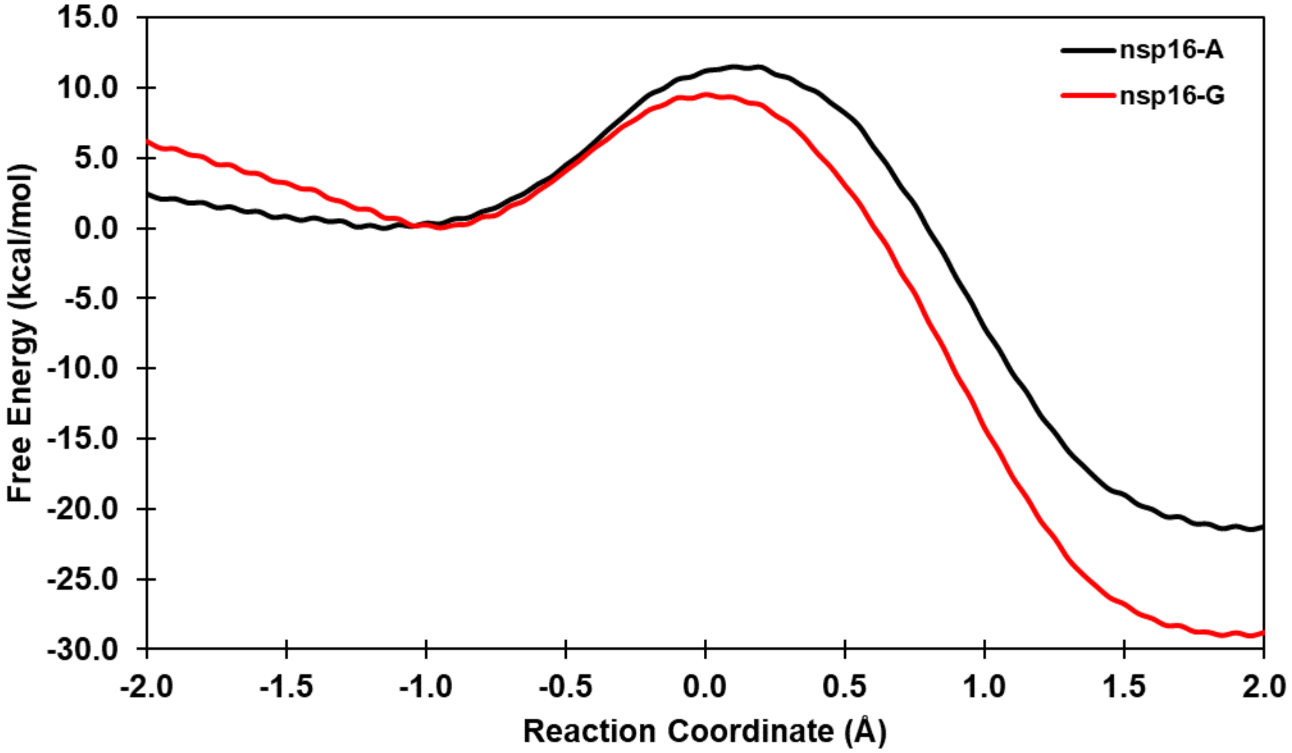

Supplementary Figure S4. Free energy profile (in kcal·mol^{-1}) for the S_N2 mechanism at DFTB3/MM level forthe nsp16-A (black) and nsp16-G (red) systems.

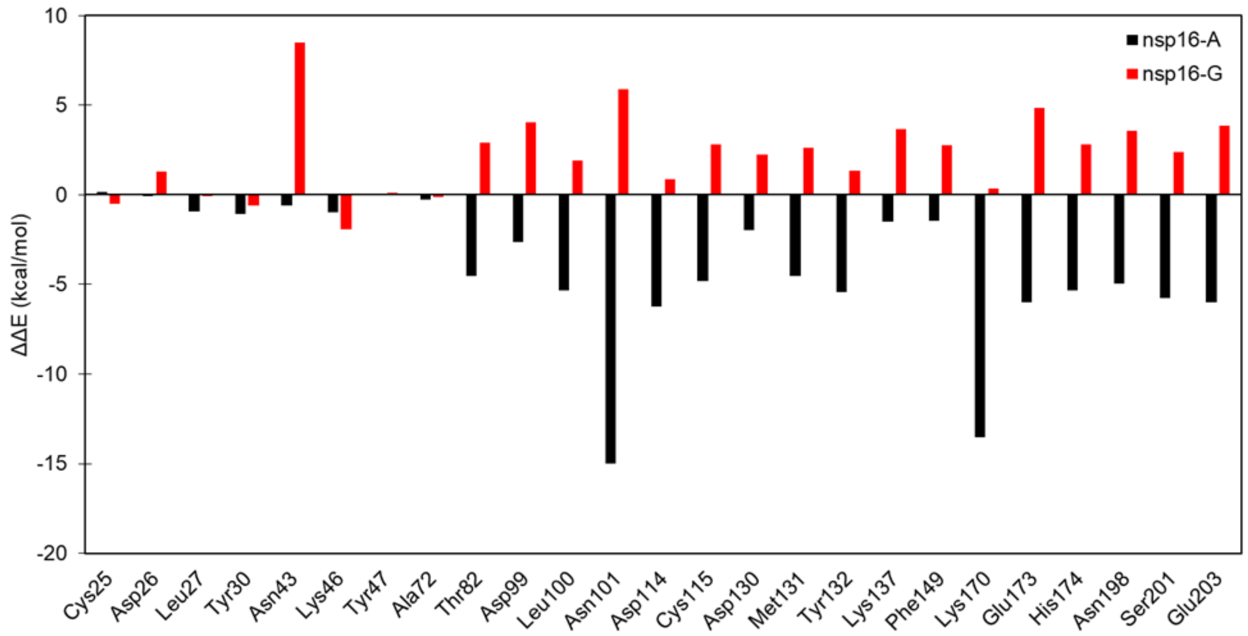

Supplementary Figure S5. Relative stabilization pattern of the most relevant active site residues on thensp16-A (black) and nsp16-G (red) systems at DFTB3/MM level.
